# Effect of Vacuum and Modified Atmosphere Packaging on the Shelf Life and Quality of Gutted Rainbow Trout (*Oncorhynchus mykiss*) during Refrigerated Storage

**DOI:** 10.3390/foods12163015

**Published:** 2023-08-10

**Authors:** Jelena Babic Milijasevic, Milan Milijasevic, Slobodan Lilic, Jasna Djinovic-Stojanovic, Ivan Nastasijevic, Tamara Geric

**Affiliations:** Institute of Meat Hygiene and Technology, Kaćanskog 13, 11000 Belgrade, Serbia

**Keywords:** rainbow trout, psychrotrophic bacteria, *Enterobacteriaceae*, TBARS, TVB-N, sensory assessment, shelf life, vacuum packaging, MAP, refrigeration

## Abstract

The quality changes of gutted rainbow trout in vacuum packaging (VP) and modified atmosphere packaging (MAP) with 40% CO_2_ + 60% N_2_ (MAP1), 60% CO_2_ + 40% N_2_ (MAP2), and 90% CO_2_ + 10% N_2_ (MAP3) were evaluated. The samples were stored at 3 ± 0.5 °C, and on days 1, 4, 7, 10, 13, and 16 of storage, microbiological, chemical, and sensory testing was performed. The aerobic plate count (APC) and psychrotrophic bacteria count (PBC) in VP fish exceeded the conventional limit of 7 log cfu/g on day 10, and in MAP1 and MAP2 fish on day 16, whereas in MAP3 fish, their number remained below that limit during the experiment. MAP significantly slowed down the growth of *Enterobacteriaceae* in trout, and the degree of inhibition increased with increasing CO_2_ concentration in the gas mixture. The lowest lactic acid bacteria numbers were detected in VP fish, whereas the highest numbers were determined in trout packaged in MAP2 and MAP3. Significantly lower numbers of hydrogen sulfide-producing (H_2_S) bacteria were detected in fish packed in MAP. Distinct patterns were observed for pH among treatments. The lowest increase in TBARS values was detected in VP and MAP3 fish, whereas in MAP1 and MAP2 fish, the TBARS values were higher than 1 mg MDA/kg on day 16 of storage when a rancid odor was detected. MAP inhibited the increase in total volatile basic nitrogen (TVB-N) content of trout compared to trout packaged in a vacuum. The sensory attributes of trout perceived by the sensory panel changed significantly in all experimental groups during storage. Based primarily on sensory, but also microbial, and chemical parameters, MAP has great potential for preserving fish quality and extending the shelf life of gutted rainbow trout from 7 days in VP to 13 days in MAP1 and MAP2, and to 16 days in MAP3.

## 1. Introduction

Rainbow trout (*Oncorhynchus mykiss*) was first introduced in Germany in 1882 and now, with common carp, it is one of the most important aquaculture fish raised in Europe. Trout production in the world and in Serbia is constantly increasing. The main trout producers are Iran (206,050 tonnes in 2019) and the European Union (EU) 27 with 183,819 tonnes; together, they account for 42% of world production. The main EU member states producing trout are France, Italy, and Denmark, accounting for 56% of EU production [1]. In the last three years, 4874 tons (live weight) of trout were produced in Serbia [2]. In Serbia, rainbow trout farms are located in the highland parts of the country, and the water supplies come from larger-sized springs or mountain streams and rivers. Today, trout is among the most sought-after species of fish in the European market because of its high quality and desirable characteristics, such as aroma, taste, and white flesh.

Modern consumers demand fresh fish for consumption rather than frozen or processed fish. This requirement is largely met by packaging the products in a vacuum or a modified atmosphere. Vacuum packaging (VP) and modified atmosphere packaging (MAP) are considered established technologies for food preservation, and they work by changing the gas proportions in a food environment by withdrawing O_2_ or by replacing the atmosphere inside the package with a mixture of gases, such as carbon dioxide (CO_2_) and nitrogen (N_2_) gas [3]. CO_2_ is the main gas used as a bacteriostatic agent against fish microbiota. Several studies have demonstrated that high CO_2_ levels extend the shelf life of fish and fish products due to the inhibition of microbial growth [4,5,6,7]. MAP with high levels of CO_2_ generally promotes fresh fish storage stability [8]. In addition, oxygen (O_2_) gas from the package is substituted with inert nitrogen (N_2_), resulting in the inhibition of the growth of aerobic microorganisms and a decrease in oxidative rancidity [9]. The action of both technologies can prolong the shelf life of fish and promote sensory attributes under refrigeration conditions, maintaining the quality of the product throughout its shelf life [10]. The evaluation of different quality parameters, such as microbial, chemical, physical, and sensory attributes, has been used to determine the shelf life of fish [11]. The shelf life of filleted rainbow trout (*S. gairdneri*) in the over-wrap package, vacuum, and MAP stored at 2 °C was evaluated by Randell et al. [12]. Based on the results of the microbial and sensory analyses, the authors concluded that trout fillets deteriorated faster in over-wrap and vacuum packages in comparison to gas packages. The freshness of rainbow trout (*O. mykiss*) stored for up to 12 days was examined in ice without gutting, in ice after gutting, and under refrigeration after gutting and vacuum packing by Rodriguez et al. [13] using sensory and biochemical parameters. The results suggested that chemical parameters hypoxanthine and K value were applicable as indicators of the freshness of trout stored in ice, regardless of whether the fish were whole or gutted. At the same time, these parameters were not relevant for vacuum-packaged gutted trout. In another study on the quality assessment of rainbow trout (*Oncorhynchus mykiss*) fillets packaged in over-wrap, vacuum, and modified atmospheres, a significant extension of the shelf life of filleted trout packaged in MAP was reported [14]. When examining the effect of vacuum packaging on physicochemical changes in eviscerated rainbow trout (*Oncorhynchus mykiss*) during cold storage, Ježek & Buhtova [15] concluded that VP effectively slowed down oxidative changes in trout muscle. On the other hand, the effects of UV-C radiation, vacuum, MAP (80% CO_2_/20% N_2_), and their combination on rainbow trout (*Oncorhynchus mykiss*) fillets quality parameters were examined during refrigeration [5]. This study demonstrated that MAP enhanced the shelf life of trout filets and that UV-C radiation did not affect it. Regarding the preservation of trout using vacuum and modified atmosphere packaging, there was generally very limited information in the literature on the effects of VP and MAP on the preservation of quality parameters of gutted rainbow trout (*Oncorhynchus mykiss*). Furthermore, in the published scientific papers, there was a lack of a significant number of studies that referred to the packaging of gutted rainbow trout (*Oncorhynchus mykiss*) in a modified atmosphere with very high concentrations of CO_2_.

Therefore, the aims of this research were to monitor changes in selected microbial, chemical, and sensory parameters of gutted rainbow trout (*Oncorhynchus mykiss*) packaged in a vacuum and modified atmosphere during storage at 3 ± 0.5 °C, and to consider the best gas mixture for extending the shelf life of packaged fish.

## 2. Materials and Methods

### 2.1. Fish Material and Experimental Design

A total number of 270 live rainbow trout (*Oncorhynchus mykiss*) with an average market size (weight 293 g/length 28 cm) were collected from a commercial fishpond with an intensive rearing system. The ponds used for raising trout were supplied with high-quality water directly from a spring with a capacity of 500 L/s. The water temperature of 13 °C was constantly maintained throughout the year. 

After collection, live fish were transported to the fish plant (slaughter and processing facility). The trout were stunned by an electric current immediately after being caught in the holding tank at the slaughter facility. Fish evisceration was performed on an automatic Baader 140 device with a capacity of 20 fish per minute. The gills were removed manually, and then the fish carcasses were washed under running water and arranged on perforated trays where they were drained for one hour.

The fish were divided into four groups. One group, packaged in a vacuum (VP fish), was used as the control. The other three groups of trout were packaged in a modified atmosphere with different gas ratios: MAP1 fish in 40% CO_2_ + 60% N_2_; MAP2 fish in 60% CO_2_ + 40% N_2_; and MAP3 fish in 90% CO_2_ + 10% N_2_. The packing machine was a Variovac (Variovac Primus, Zarrentin, Germany), and the packaging foil was OPA/EVOH/PE (oriented polyamide/ethylene vinyl alcohol/polyethylene, Dynopack, Polimoon, Kristiansand, Norway) with low gas permeability (degree of permeability for O_2_—3.2 cm^3^/m^2^/day at 23 °C, for N_2_—1 cm^3^/m^2^/day at 23 °C, for CO_2_—14 cm^3^/m^2^/day at 23 °C, and for steam—15 g/m^2^/day at 38 °C). The gas:fish ratio in all the MAP packages was 2:1. All packaged fish were stored under the same conditions at 3 ± 0.5 °C and on days 1, 4, 7, 10, 13, and 16 of storage, microbiological, chemical, and sensory testing was performed. For the VP fish, the last examination was conducted on day 10 of storage when sensory changes were revealed.

### 2.2. Gas Analyses

The gas composition in the headspace of all MAP samples was analyzed using a Headspace Gas Analyser Oxybaby V O_2_/CO_2_ (Witt-Gasetechnik GmbH & Co KG, Witten, Germany) by penetrating a needle through a gas-tight septum prior to opening the packages. Measurements were conducted before each sampling.

### 2.3. Microbiological Analysis

For each of the four packaging conditions, trout from three packages were analyzed on each sampling day. For the analyses, from each package, 20 g of fish muscle was removed aseptically with a sterile scalpel and tweezers, transferred into a stomacher bag, diluted with 180 mL of 0.9% NaCl solution, and homogenized for 2 min (Steward Stomacher 400 Lab Blender, London, UK). For all microbial enumerations, ten-fold serial dilutions were prepared and 1 mL from each dilution was used.

Aerobic plate count (APC) and psychrotrophic bacteria count (PBC) were determined on plate count agar (PCA, Merck, Darmstadt, Germany) using the pour plate technique with agar tempered to 44 °C. Plates were incubated at 30 °C for 72 h and 6.5 °C for 10 days.

To determine the *Enterobacteriaceae* count, 1 mL of suitable dilutions was inoculated into 10 mL of molten and tempered (44 °C) violet red bile glucose agar (VRBGA, Merck, Darmstadt, Germany). After the substrate solidified, an additional overlay of 15 mL of molten VRBGA was added to prevent overgrowth and generate semi-anaerobic conditions. Plates were incubated at 37 °C for 24 h.

Lactic acid bacteria (LAB) were enumerated on de Man, Rogosa, and Sharp agar (MRS, Merck, Darmstadt, Germany). Volumes (1 mL) of suitable dilutions were poured into 15 mL of molten and tempered (44 °C) agar. Plates were incubated at 30 °C for 3 days.

For enumeration of hydrogen sulfide-producing (H_2_S) bacteria, 1 mL of suitable dilutions was poured into 15 mL of molten and tempered (44 °C) iron agar (Merck, Darmstadt, Germany). After the substrate solidified, a 10 mL overlay of molten agar was added. The iron agar plates were incubated at 37 °C for 24 to 48 h under anaerobic conditions (Gas Pak Anaerobic System, bioMérieux, Craponne, France).

All plates were prepared in duplicate and examined visually for typical colony types and morphological characteristics, and counts were expressed as logarithms of the number of colony-forming units per gram (log cfu/g).

### 2.4. Chemical Analysis

#### 2.4.1. pH

After homogenization of a suitable amount of fish muscle, the pH was measured using Cyber Scan pH-510 digital pH-meter (EUTECH Instruments, Breda, The Netherlands).

#### 2.4.2. Lipid Oxidation

Lipid oxidation, measured as 2-thiobarbituric acid reactive substances (TBARS), was determined in triplicate using the distillation method according to Tarladgis, Pearson and Dugan, 1964 [16] and Holland, 1971 [17], and oxidation products were quantified as malondialdehyde (mg/kg MDA). Briefly, an appropriate quantity of distilled water was added to 20 g of homogenized fish muscle, and HCl (4 M) was added to adjust the pH to 1.5. The acidified solutions were distilled at room temperature. Subsequently, 5 mL of the distillate was added to 5 mL of TBA solution (0.3%, *m*/*v*), heated in a water bath at 100 °C for 30 min, and cooled. The intensity of the red-colored solution was measured immediately on a Genesys 10S UV-VIS spectrophotometer (Thermo Fisher, USA) at a wavelength of 532 nm. Calibration curves, Y = −0.0012 + 1.042X, were generated using a standard solution of 1,1,3,3-tetraetoksi propane in ethanol at concentration levels ranging from 0.2 to 1 mL^−1^. The malondialdehyde content, expressed as MDA (mg kg^−1^), was calculated as follows: c × 100/m, where c is the concentration of MDA (µg) from the calibration curve and m is the mass of sample (g) for analysis.

#### 2.4.3. Total Volatile Basic Nitrogen (TVB-N)

The TVB-N was determined in triplicate, in accordance with the method defined in Regulation (EC) No 2074/2005 [18], using the official steam distillation. The results are expressed as mg TVB-N/100 g. Briefly, perchloric acid solution (6%) was added to 10 g of homogenized fish muscle, and after extraction, it was subjected to homogenization, filtration, and distillation after neutralization with NaOH solution. In the end, the adsorbed alkaline compounds were titrated with a standard solution of HCl (0.01 M) to pH 5.0 ± 0.1.

Chemical analysis was prepared in triplicate.

### 2.5. Sensory Assessment

To evaluate the freshness of the trout, a numerical evaluation of the quality parameters was used. The selection of quality attributes and parameters was based on the sensory evaluation of chilled fish as proposed in Regulation (EEC) No 103/76 [19], but these were somewhat modified. Sensory attributes were evaluated using the Quality Index (QI) protocol with four quality attributes and nine parameters (Table 1). The parameters received scores ranging from 0 to 2 and from 0 to 3, depending on the characteristics. The sum of the protocol scores was 22 demerit points, comprising 4 points for the eyes, 6 for the flesh, 9 for the skin, and 3 for the abdomen. Furthermore, six experienced evaluators and laboratory staff in the department for sensory evaluation, with more than 10 years of experience, conducted the sensory analysis (at a room temperature of 20 °C and under adequate lighting). Fish were removed from the packaging 10 min before analysis and presented separately on trays coded with randomly chosen 3-digit numbers. Each panelist analyzed the fish individually and recorded the score for each parameter in the QI protocol.

### 2.6. Statistics

For microbiological, chemical, and sensory data, the mean values and standard deviations were determined by processing six values for each analyzed group using column statistics. Group mean values are presented in the results section. One-way ANOVA was used to determine significant differences between the groups. When a significant F value was found, additional post hoc tests with Tukey’s adjustment were performed. Statistical significance was set at the (*p* < 0.05) level of confidence. All analyses were performed using the program Microsoft Office Excel 2016 “16.0”.

## 3. Results and Discussion

### 3.1. Gas Composition Analysis

During the storage, changes in the composition of gas mixtures were recorded for all examined groups. Initially, the CO_2_ content in MAP1 packages was 39%, in MAP2 packages 60%, and in MAP3 packages 91%. Throughout the storage period, CO_2_ dissolved into the product, which led to a decrease in its concentration in all the MAP packages. At the end of the experiment, CO_2_ content was reduced to around 34% for MAP1 samples, 53% for MAP2, and 81% for MAP3 samples. 

At the beginning of the experiment, the content of residual oxygen in all MAP groups was ≤0.5%, and during the storage never exceeded 1%. 

### 3.2. Microbiological Analysis

The initial low numbers of mesophilic and psychrotrophic bacteria (Table 2) imply good microbial quality of the fish. The trout used were grown in a fishpond supplied with a fresh spring of running water, which could be a reason for these low microbial counts. Good hygiene practices during the growing and handling of the fish could also help limit bacterial contamination, as inferred from these findings.

An increase in the APC during the storage period was observed in all types of packaging, with the most exceptional changes occurring in the control VP fish, which were significantly higher (*p* < 0.05) from day 4 of storage compared to MAP fish. From day 4, the APC was significantly lower (*p* < 0.05) in the group with the highest percentage of CO_2_, and this trend was observed until the end of storage. Nevertheless, no significant differences (*p* > 0.05) were noticed between MAP1 and MAP2 fish until day 10 when fish packaged in a gas mixture with 60% CO_2_ had lower APCs.

During the storage, PBC increased similarly to that of APC. The PBC increase was the most pronounced in VP fish, and the lowest PBC was detected in MAP3 fish packed in a gas mixture composed of 90% CO_2_ + 10% N_2_. 

Our results indicated a strong bacteriostatic effect of CO_2_ and a consequent reduction in the growth of mesophilic and psychrotrophic bacteria during the entire storage period. This effect depended on the applied CO_2_ concentration and is in agreement with numerous results from the literature, particularly with regard to monitoring the changes in psychrotrophic aerobic Gram-negative bacteria, which are the main cause of spoilage in raw food rich in proteins, such as fish. The results of Provincial et al. [4] indicated that there was a significant reduction in the aerobic plate count in fresh sea bass (*Dicentrarchus labrax*) fillets packaged in a gas mixture with high CO_2_ content compared to control fillets. Similar results were published by Rodrigues et al. [5], who investigated the shelf life of trout (*Oncorhynchus mykiss*) fillets packaged in MAP and in MAP with applied UV-C radiation, and then Esteves et al. [6], who investigated gray triggerfish (*Balistes capriscus*) fillets packaged in vacuum and in MAP, as well as Zhang et al. [20], who examined golden pompano (*Trachinotus ovatus*) stored under different packaging conditions.

In our study, a faster growth of PBC compared to APC at the end of the study was observed. This could be explained by the storage temperature, which was close to the ideal temperature for PBC growth. The same results were observed in salmon (*Salmo salar*) fillets packaged in a modified atmosphere [21] and in sea bass (*Dicentrarchus labrax*) fillets [4].

According to the ICMSF [22] recommendations, the aerobic plate count (APC) should not exceed 7 log cfu/g in fresh fish. This limit was also considered a microbiological criterion that indicated the shelf life of fish during storage. In our study, in VP fish, APC reached numbers above the recommended limit on day 10, and in MAP1 and MAP2 fish on day 16; this corresponded with sensory unacceptability, i.e., spoilage. In MAP3 fish, APC never exceeded 7 log cfu/g over the 16 days of the study. 

Our research showed that there was a good correlation between the aerobic plate counts of trout packaged in vacuum and modified atmospheres and the trout’s overall sensory acceptability (see below). Research conducted by Fletcher et al. [23] indicated that the aerobic plate count in salmon (*Oncorhynchus tshawytscha*) fillets packaged in a modified atmosphere (40% CO_2_ + 60% N_2_) was a good indicator of fish spoilage. In salmon (*Salmo salar* L.) fillets packaged in a modified atmosphere (60% CO_2_ + 40% N_2_) or vacuum, Hansen et al. [24] also found a high level of correlation between APC and odor, the main sensory attribute of fish freshness, which is in agreement with our study.

On the other hand, Özogul and Özogul [25] in their research found a weak correlation between APC and sensory acceptability of fresh sardines (*Sardina pilchardus*) packaged in a gas mixture composed of 60% CO_2_ and 40% N_2_, as well as in vacuum and air, considering that the aerobic plate count reached maximum levels while the fish were still acceptable from a sensory point of view. The same result was published by Stamatis and Arkoudelos [26], who proved that the APC did not exceed 7 log cfu/g at the time of the first signs of spoilage of fresh sardine (*Sardina pilchardus*) fillets packed in a modified atmosphere. 

At the beginning of our study, low numbers of *Enterobacteriaceae* were found in all the fish groups, suggesting good hygiene and handling practices. As expected, *Enterobacteriaceae* increased as the storage time progressed (*p* < 0.05). The highest numbers of *Enterobacteriaceae* were detected in VP fish, whereas the lowest numbers were detected in fish packaged in the modified atmosphere with the highest concentration of CO_2_ (MAP3). During the first seven days of the study, no significant differences (*p* > 0.05) were found between the numbers of *Enterobacteriaceae* in MAP1 and MAP2 fish. This could be explained by the fact that at the beginning of the study, concentrations of 40% and 60% CO_2_ were equally effective in inhibiting these microorganisms. After the first 7 days and until the end of storage, the number of *Enterobacteriaceae* was significantly lower (*p* < 0.05) in the group packaged with 60% CO_2_. Our research showed that MAP significantly slowed down the growth of *Enterobacteriaceae* in trout, and the degree of inhibition increased with an increase in CO_2_ concentration in the gas mixture. 

The antimicrobial effect of CO_2_ is indicated by Milijašević et al. [7], who found a significantly lower number of *Enterobacteriaceae* in carp (*Cyprinus carpio*) steaks packaged in 100% CO_2_ compared to steaks packaged in a gas mixture (40% CO_2_ + 60% N_2_) after 15 days of storage at 3 °C. Also, Stamatis and Arkoudelos [26] suggested that CO_2_ concentration is a very important growth-inhibiting factor for *Enterobacteriaceae* in sardine (*Sardina pilchardus*) fillets packed in a modified atmosphere (50% CO_2_ + 50% N_2_). Furthermore, other storage studies at 3 °C showed that CO_2_ in higher concentrations inhibits the growth of *Enterobacteriaceae* in trout (*Oncorhynchus mykiss*) and carp (*Cyprinus carpio*) cuts [27], smoked trout (*Oncorhynchus mykiss*) fillets [28], and hake (*Merluccius merluccius*) steaks [29]. The results of our research are in agreement with the results of the abovementioned authors, who concluded that CO_2_ in the packaging has a protective effect on the fish, leading to reductions in the aerobic plate count and *Enterobacteriaceae*.

In all our experimental groups, the total number of LAB increased during storage. The lowest LAB numbers were detected in VP fish, while the highest numbers were determined in trout packaged in gas mixtures with higher percentages of CO_2_ (MAP2 and MAP3). In these fish, the number of LAB exceeded 7 log cfu/g on day 16 of storage, whereas in the MAP1 fish, with 40% CO_2_, LAB reached 6.63 ± 0.13 log cfu/g. The faster growth of LAB under MAP conditions compared to other bacterial species could be explained by the tolerance of LAB to CO_2_ [30] and by the ability of LAB to inhibit the growth of other microorganisms by producing lactic acid and bacteriocins [26]. 

Babić Milijašević [31] also stated that high concentrations of CO_2_ (70 to 100%) lead to the development of dominant heterofermentative *Lactobacillus* microbiota in packaged fish. Research conducted by Stenstrom [32] indicated that the microbiota of fresh fish is composed of 62 to 85% of LAB, which takes part in the process of spoilage of cod fillets packed in a mixture of gases with high CO_2_ concentrations (90 to 100%). This could be explained by the fact that Gram-negative aerobic psychrotrophic rod-shaped bacteria, which make up the largest part of the normal microbiota of fish, are largely inhibited under anaerobic conditions, while the growth of facultatively anaerobic bacteria such as LAB is stimulated under the mentioned conditions [33].

On the contrary, Lalitha et al. [34] revealed no increase in the number of LAB in Asian perch (*Etroplus suratensis Bloch*) packaged in a modified atmosphere with different percentages of CO_2_ (40 to 70%), and these microorganisms had no important role in fish spoilage. An investigation conducted by Masniyom et al. [35] also indicated a lower number of LAB in sea bass (*Lates calcalifer*) packaged in 100% CO_2_ compared to fish packaged in modified atmospheres with 60 to 80% CO_2_. These authors concluded that 100% CO_2_ had an inhibitory effect on the growth of not only aerobic bacteria that caused meat spoilage but also LAB.

In the present study, at the beginning of storage, the number of H_2_S-producing bacteria was low, indicating that these bacteria constitute only a minor fraction of the microbiota in freshly slaughtered fish. During storage, increases in the number of H_2_S-producing bacteria were affected by the gas atmosphere used. The highest numbers of H_2_S-producing bacteria were detected in VP fish, which could be explained by the ability of these microorganisms to use trimethylamine oxide (TMAO) as the final electron acceptor in anaerobic respiration [36]. Significantly lower numbers of H_2_S-producing bacteria were detected in fish packed in MAP, particularly in those fish with the highest CO_2_ concentration. When APC reached the limit of 7 log cfu/g in MAP1 and MAP2 fish, the number of H_2_S-producing bacteria was around 6 log cfu/g, demonstrating that H_2_S-producing bacteria probably did not take part in forming the unpleasant spoilage odor. Many bacteria from the H_2_S-producing bacteria, including *Shewanella putrefaciens*, do not have the ability to grow in low O_2_ together with high CO_2_ concentrations [37]. These results correspond to others showing that H_2_S-producing bacteria are inhibited by CO_2_ gas in Atlantic salmon (*Salmo salar*) [38] and seer fish (*Scomberomorus commerson*) [39].

### 3.3. Chemical Analysis

**Figure 1:**
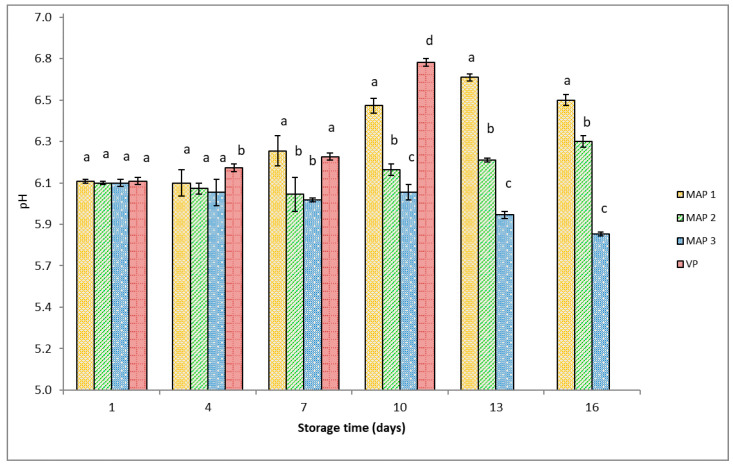
pH of gutted rainbow trout packaged under different conditions during storage at 3 °C. Packaging type: MAP1 (40% CO_2_ + 60% N_2_), MAP2 (60% CO_2_ + 40% N_2_), MAP3 (90% CO_2_ + 10% N_2_), and VP (vacuum packaging). Bars indicate standard error. Different lowercase letters indicate significant differences (*p* < 0.05) in means (n = 3) between packaging systems on the same day.

Figure 1 shows the pH of gutted rainbow trout packaged in different atmospheres during storage. The initial pH of trout muscle on day 1 was 6.11 ± 0.02 (mean ± SD). In VP and MAP1 fish, a significant (*p* ˂ 0.05) increase in pH was observed between day 7 (6.24 ± 0.02 and 6.27 ± 0.08, respectively) and day 10 (6.63 ± 0.02 and 6.51 ± 0.04, respectively) of the study. From then on to the end of storage, the pH of the fish in MAP1 fluctuated. In contrast, the pH of MAP2 and MAP3 fish decreased during the whole storage period. Compared to MAP2 fish, MAP3 fish had a lower pH from day 10 of storage. The mean pH values for trout packaged in VP, MAP1, MAP2, and MAP3 during storage were 6.32 ± 0.02, 6.36 ± 0.04, 6.15 ± 0.03, and 6.00 ± 0.02, respectively.

The lowest pH was observed in trout packed with 90% CO_2_ (Figure 1). Some researchers found a significantly lower pH in fish packed in a modified atmosphere with a high percentage of CO_2_ [4,6,7], which is in agreement with the results of our research. This is due to the dissolution of CO_2_ within the fish muscle tissue and the consequent accumulation of carbonic acid (H_2_CO_3_). However, Stenstrom [32] concluded that the decrease in pH can be explained by the accumulation of acid metabolic products created by various types of bacteria, primarily LAB. 

The pH slightly increased in the fish packed in VP and MAP1 after 7 days of storage. This can be explained by the accumulation of basic substances that are the products of the metabolism of fish spoilage bacteria [40]. These bacteria had more favorable conditions for growth in VP and MAP1 due to the lower concentrations of CO_2_ in the packages than in our higher CO_2_ MAP (MAP2 and MAP3). In our study, the pH of trout muscle, including the pH differences under the different experimental conditions, encountered during storage corresponded to the observations from other studies [35,41,42]. In contrast, Arashisar et al. [43] did not find significant differences in the pH of rainbow trout (*Oncorhynchus mykiss*) fillets packaged under different atmosphere conditions.

**Figure 2:**
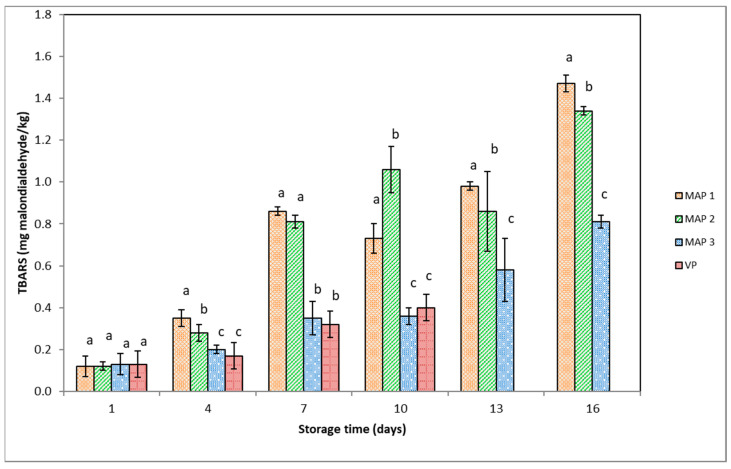
TBARS values of gutted rainbow trout packaged under different conditions at 3 °C during storage. Packaging type: MAP1 (40% CO_2_ + 60% N_2_), MAP2 (60% CO_2_ + 40% N_2_), MAP3 (90% CO_2_ + 10% N_2_), and VP (vacuum packaging). Bars indicate standard error. Different lowercase letters indicate significant differences (*p* < 0.05) in means (n = 3) between packaging systems on the same day.

The TBARS values of the gutted rainbow trout stored under vacuum and modified atmospheres are shown in Figure 2.

An increase in TBARS values in all fish groups was detected during the storage time period (*p* < 0.05). The lowest increase was detected in VP and MAP3 fish, with no differences (*p* > 0.05) in TBARS values. Throughout the storage period, the TBARS values in MAP1 and MAP2 fish were higher than those in VP and MAP3 fish (*p* < 0.05). In these two former groups during the storage, only slight fluctuation was found in the TBARS values. Considering that the TBARS value is a measure of MDA content that is not stable over a long period of time, the fluctuation in the TBARS values in MAP1 and MAP2 fish could have been caused by the interaction between MDA and proteins, amino acids, glycogen, etc., leading to a lower content of free MDA [44].

It is interesting to note that although the MAP gases in our study did not contain O_2_, significant quantities of 2-thiobarbituric acid-reactive substances were found in this packaging type. This suggests that the production of MDA does not depend solely on the amount of O_2_ present in the package but is rather multi-factorial. This is probably linked to the inactivation of antioxidative enzymes and subsequent production of carbonic acid in fish muscle, in packages with high CO_2_ concentrations [35], the type of microbiota present, including the CO_2_ dissolved in the tissue that favors polyunsaturated fatty acid autoxidation [40]. Masniyom et al. [35] reported similar observations for sea bass (*Lates calcalifer*) packaged in the presence of air in all MAP, as well as control fish samples, where lipid oxidation was recorded in sea bass even though they were stored in 100% CO_2_. The authors pointed out that sea bass stored in 60% or 80% CO_2_ atmospheres had higher TBARS values than the control air-packaged fish.

Connell [45] concluded that TBARS of 1–2 mg MDA/kg of fish flesh is usually considered the threshold limit beyond which fish will normally develop an unfavorable odor or taste. In the present study, the TBARS values did not exceed 1 mg MDA/kg during the storage for VP fish (0.47 mg MDA/kg after 10 days of storage) and MAP3 fish (0.81 mg MDA/kg after 16 days of storage). In contrast, the TBARS values of MAP1 and MAP2 fish exceeded the limit of 1 mg MDA/kg (1.47 and 1.34 mg MDA/kg, respectively) on day 16 of storage. 

Since a rancid odor was detected in MAP1 and MAP2 fish when the TBARS values were higher than 1 mg MDA/kg, we suggest that this level could be considered a limit above which changes in odor occur in trout muscle. Also, CO_2_-rich packaging effectively inhibited microbial spoilage but did not prevent chemical changes, especially lipid oxidation. For this reason, some antioxidants would have to be applied in combination with MAP to achieve an extended shelf life in terms of microbial and chemical changes.

**Figure 3:**
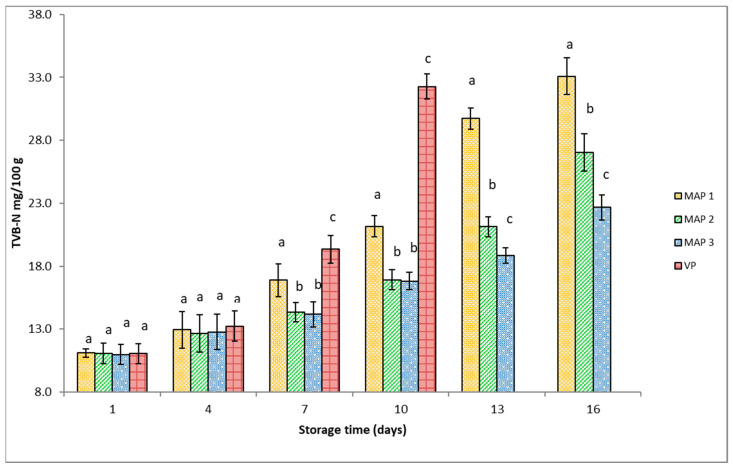
TVB-N values (mg/100 g) of gutted rainbow trout packaged under different conditions at 3 °C during storage. Packaging type: MAP1 (40% CO_2_ + 60% N_2_), MAP2 (60% CO_2_ + 40% N_2_), MAP3 (90% CO_2_ + 10% N_2_), VP (vacuum packaging). Bars indicate standard error. Different lowercase letters indicate significant differences (*p* < 0.05) in means (n = 3) between packaging systems on the same day.

Figure 3 shows TVB-N values (mg/100 g) of gutted rainbow trout during the storage period. At the beginning of storage, the TVB-N values of the trout were practically identical (*p* > 0.05). The concentrations of these degradation products in the MAP fish remained mostly unchanged until day 4 of storage. From then on, the TVB-N values increased in all fish groups during the storage period. As shown in Figure 2, TVB-N values in the trout were strongly influenced by the packaging atmosphere. Increases in TVB-N values were in the following order: MAP3 < MAP2 < MAP1 < vacuum, ranging from 10.98 ± 0.80 to 22.66 ± 0.98 mg N/100 g in the MAP3 fish, from 11.07 ± 0.82 to 27.04 ± 1.50 mg N/100 g in the MAP2 fish, and from 11.10 ± 0.35 mg N/100 g to 33.10 ± 1.45 mg N/100 g in the MAP1 fish during the 16 day storage period; for VP fish, TVB-N values ranged from 11.05 ± 0.80 to 32.28 ± 0.84 mg N/100 g during the 10 day storage period. During this study, TVB-N levels in MAP3 fish did not change as much as those in MAP1, MAP2, and VP fish. Starting on day 7, TVB-N values of MAP2 and MAP3 fish were considerably lower (*p* < 0.05) than the TVB-N values of MAP1 and VP fish.

Interestingly, within the first 10 days of storage, no statistically significant differences (*p* > 0.05) were observed in TVB-N values between MAP2 and MAP3 fish. This could be attributed to the fact that the gas mixture of 60% CO_2_ + 40% N_2_ is sufficient to inhibit microbial activity, as shown by the low TVB-N values. A higher concentration of CO_2_ is likely required to inhibit microbial activity and subsequent spoilage once microbial populations increase.

In our research, trout stored under a high concentration of CO_2_ showed low TVB-N values, and generally, the VP fish had higher TVB-N values compared to the CO_2_-enriched MAP fish throughout the storage period.

In a study carried out by Masniyom et al. [46], high CO_2_ concentrations showed the potential to inhibit the growth of mainly Gram-negative microorganisms and decrease the deamination capacity of bacteria, which led to the lower production of volatile compounds. The same observations were reported by Milijašević et al. [7] and Babić et al. [47] for carp (*Cyprinus carpio*) and by Zhang et al. [20] for golden pompano (*Trachinotus ovatus*) stored under MAP, supporting the results of the present study. 

Some studies reported the threshold values for TVB-N with a limit of 25 to 35 mg N/100 g as a recommendation for rejecting commercial fresh whole fish and processed fish products [45]. Given that no regulatory limit is defined regarding the acceptability of rainbow trout by the Regulation (EC) 2074/2005 [18], Arashisar et al. [43] recommended 25 mg N/100 g as the threshold value for TVB-N in trout (*Oncorhynchus mykiss*) muscle. In our study, the TVB-N values of MAP3 fish mainly remained constantly below this limit throughout the storage period. The only event when this limit was exceeded was observed in VP fish (day 10) and in MAP1 and MAP2 fish (day 16).

### 3.4. Sensory Assessment

**Figure 4:**
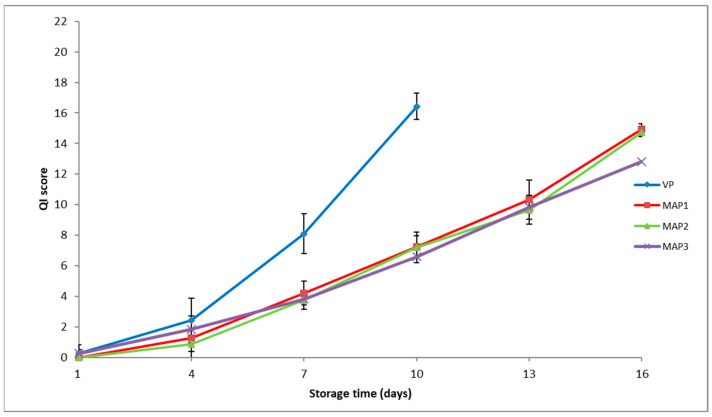
Quality index (QI) score of gutted, rainbow trout packaged under different conditions during storage at 3 °C. Packaging type: MAP1 (40% CO_2_ + 60% N_2_), MAP2 (60% CO_2_ + 40% N_2_), MAP3 (90% CO_2_ + 10% N_2_), and VP (vacuum packaging). Bars indicate standard error.

As shown in Figure 4, QI scores increased over time in all experimental groups. At the beginning of storage (day 1), the quality of the fish was characterized by a uniform light rosy color and elastic texture of flesh, bright and pearly shine to the skin with transparent mucus, fresh, neutral odor, and black convex pupils. QI scores did not change significantly until day 4, so on days 1 and 4, fish from all groups were assessed as fresh and of very good quality. From day 4 onwards, as expected, sensory parameters changed differently under the different packaging conditions.

The greatest increase in all QI scores was observed in the VP fish. A closer analysis showed that the attributes that had the greatest impact on the increase in QI scores in VP fish were the odor of the abdomen and skin, flesh color, and shape of eyes. The odor of VP fish changed faster than that of MAP fish. At the moment of sensory rejection, the odor of VP fish was sour and unpleasant. Various odorous sulfur compounds, together with volatile fatty acid and ammonia, which are produced during bacterial growth, as well as the accumulation of secondary lipid oxidation products, such as aldehydes, ketones, and carbonyls [9], could be a reason for off-odor development and discoloration. The negative impact of VP could have caused higher scores for eye shape in this group. After 10 days of storage in VP, the QI score reached the limit of acceptability of 16.42, when panelists rejected the fish as unsuitable for consumption from a sensory point of view. This value accounted for around 75% of the demerit points in our QI protocol.

In the MAP1 and MAP2 fish, increases in QI scores were slower compared to VP. During storage, slight but significantly lower (*p* < 0.05) QI values were recorded for MAP2 fish compared to MAP1 fish from day 10 onwards. In MAP1 and MAP2 fish, the sensory attributes that most influenced the increase in QI scores were the odor of the abdomen and skin and flesh consistency. The odor changed from that of fresh fish at the start; with extended storage, the odor became neutral to ammoniacal, while at the end of the shelf life, the odor was unpleasant, sour, reminiscent of fermentation, and rancid. The metabolic activities of microorganisms at the end of the storage period could be a reason for the unpleasant odor of fermentation, as well as for the slightly soft flesh consistency, whereas the higher degree of lipid oxidation in MAP1 and MAP2 fish could be reflected by the rancid off-odor. Panelists rejected MAP1 and MAP2 fish on day 16, whereas the QI score in MAP1 fish was 14.92 and in MAP2 fish was 14.75, i.e., around 68% and 67% of demerit points. 

The lowest QI scores for all attributes were obtained for MAP3 fish. In MAP3 fish on the last day of storage, no changes in odor were found that would have had a negative impact on the shelf life, and these fish were evaluated as acceptable from a sensory point of view. However, in this group, the increase in the QI score was most influenced by the color and consistency of the fish muscle. The reason for this could be the percentage of CO_2_ in this packaging type. By dissolving in the aqueous phase of the fish muscle, the CO_2_ in the packages led to a drop in the pH and consequent loss of meat juice, which negatively affected the consistency of the fish flesh. Also, due to the lack of O_2_ and the high percentage of CO_2_ in the gas mixture, a color with a grayish hue appeared, which was rated with lower grades by the evaluators. At the end of the storage period, the QI score of MAP3 fish was 12.83, i.e., around 58% of the demerit points. 

Therefore, evaluators rejected the VP fish on day 10, whereas MAP1 and MAP2 fish were rejected on day 16. These changes corresponded well with the quality deterioration evaluated using chemical and microbial analyses. In our study, the high bacterial load that developed during storage contributed significantly to defining the shelf life of fish; according to Gram and Huss [36], sensory characteristics (changes in odor, color, appearance, texture, and flavor) are strongly correlated with the bacterial count and are manifested as a quality loss.

However, our results demonstrate that quantitative analysis of simple bacteria groups is not sufficient to determine spoilage development more closely. In future studies, the identification of the initial bacteria in fresh trout is needed in order to fully understand the spoilage potential and mechanisms during storage under different atmosphere conditions.

## 4. Conclusions

Based on sensory, microbial, and chemical parameters, MAP has great potential to preserve fish quality and extend the shelf life of gutted rainbow trout from 7 days in VP to 13 days in MAP1 (40% CO_2_ + 60% N_2_) and MAP2 (60% CO_2_ + 40% N_2_), and to 16 days in MAP3 (90% CO_2_ + 10% N_2_). MAP with high CO_2_ concentrations produced the most desirable microbial and sensory results, and lower pH and lipid oxidation values. Hence, a study demonstrated that MAP3 (90% CO_2_ + 10% N_2_) provides a better quality of gutted rainbow trout when an extended storage period is required. The results of our experiment, in contrast to the majority of published papers in the literature, show that the ideal gas mixture for packing gutted fish, in this case rainbow trout, is one that contains 90% carbon dioxide. These results provide useful information about the storage of gutted rainbow trout under different MAP conditions, which could be useful at the processor and retailer levels in terms of minimizing waste through spoilage in fishery products.

Future research may include a combination of VP and MAP of gutted trout with pre-treatments, for instance, applying preservatives such as sodium acetate or potassium sorbate, light salting or brining of fish, or application of essential oils with antimicrobial and antioxidant effects. 

Another approach to increasing shelf life could be to incorporate or coat packaging films with substances that can slow down or inhibit microbial, enzymatic, and oxidative changes in fish by releasing active components at a controlled rate either into the inner environment or directly onto the food (known as active packaging).

## Figures and Tables

**Table 1:** Quality index (QI) method for assessing freshness of gutted trout packaged in a vacuum or modified atmosphere.

Quality Attributes	Parameter		Score
Eyes	Pupils	Black, bright with metallic radiancy	0
		Black dull	1
		Opaque grey	2
	Shape	Convex	0
		Flat	1
		Concave in the centre	2
Flesh	Color	Light rosy, smooth, shining	0
		Velvety, waxy, dull, slightly changed	1
		Slightly opaque	2
		Opaque	3
	Consistency	Firm and elastic, smooth surface	0
		Less elastic	1
		Less elastic, slightly soft, dull surface	2
		Soft, inclining to mealy	3
Skin	Color	Bright, pearly shine all over the body	0
		Less pearly shine	1
		Yellowish predominantly on the abdomen	2
	Mucus	Aqueous, transparent	0
		Slightly cloudy, milky	1
		Yellowish, opaque	2
	Odor	Fresh, neutral, typical of fish	0
		Metallic, on cucumber, on hay	1
		Rancid	2
		Unpleasant (rancid and rotten)	3
	Texture	Firm, elastic	0
		Finger mark disappears rapidly	1
		Soft, finger mark delays disappear	2
Abdomen	Odor	Fresh, neutral	0
		On cucumber, on melon	1
		Rancid, on fermentation	2
		Putrid	3
Total QI score			0–22

**Table 2:** Aerobic plate count (APC), psychrotrophic bacteria count (PBC), lactic acid bacteria (LAB), Enterobacteriaceae (ENT), and hydrogen sulfite-producing (H_2_S) bacteria expressed as log cfu/g (mean ± SD) of gutted rainbow trout in different packaging types (VP and MAP).

Parameter	Packaging Type	Days of Storage					
		1	4	7	10	13	16
APC	40% CO_2_ + 60% N_2_	2.18 ± 0.80 ^a^	3.27 ± 0.31 ^a^	4.99 ± 0.44 ^a^	6.38 ± 0.14 ^a^	6.77 ± 0.43 ^a^	7.76 ± 0.56 ^a^
	60% CO_2_ + 40% N_2_	2.20 ± 0.21 ^a^	3.14 ± 0.33 ^a^	4.77 ± 0.24 ^a^	5.84 ± 0.50 ^b^	6.59 ± 0.21 ^b^	7.28 ± 0.71 ^b^
	90% CO_2_ + 10% N_2_	2.18 ± 0.34 ^a^	2.76 ± 0.35 ^b^	3.52 ± 0.45 ^b^	4.25 ± 0.32 ^c^	4.98 ± 0.55 ^c^	5.79 ± 0.36 ^c^
	Vacuum	2.24 ± 0.34 ^a^	3.94 ± 0.56 ^c^	6.38 ± 0.32 ^c^	7.63 ± 0.26 ^d^	ne	ne
PBC	40% CO_2_ + 60% N_2_	2.68 ± 0.52 ^a^	3.88 ± 0.09 ^a^	5.06 ± 0.20 ^a^	5.91 ± 0.33 ^a^	7.68 ± 0.32 ^a^	8.39 ± 0.18 ^a^
	60% CO_2_ + 40% N_2_	2.70 ± 0.52 ^a^	3.92 ± 0.22 ^a^	4.97 ± 0.10 ^a^	6.06 ± 0.10 ^a^	7.28 ± 0.22 ^b^	8.79 ± 0.32 ^b^
	90% CO_2_ + 10% N_2_	2.68 ± 0.20 ^a^	3.13 ± 0.32 ^b^	4.09 ± 0.26 ^b^	4.94 ± 0.20 ^b^	5.58 ± 0.19 ^c^	6.50 ± 0.26 ^c^
	Vacuum	2.66 ± 0.30 ^a^	4.89 ± 0.20 ^c^	6.65 ± 0.13 ^c^	8.25 ± 0.13 ^c^	ne	ne
ENT	40% CO_2_ + 60% N_2_	1.84 ± 0.13 ^a^	2.79 ± 0.54 ^a^	3.52 ± 0.20 ^a^	4.77 ± 0.11 ^a^	5.07 ± 0.32 ^a^	5.45 ± 0.53 ^a^
	60% CO_2_ + 40% N_2_	1.86 ± 0.17 ^a^	2.74 ± 0.16 ^a^	3.25 ± 0.81 ^a^	3.94 ± 0.17 ^b^	4.16 ± 0.41 ^b^	4.99 ± 0.20 ^b^
	90% CO_2_ + 10% N_2_	1.86 ± 0.27 ^a^	2.40 ± 0.20 ^b^	2.88 ± 0.24 ^b^	3.10 ± 0.52 ^c^	3.71 ± 0.24 ^c^	4.25 ± 0.28 ^c^
	Vacuum	2.02 ± 0.25 ^a^	3.41 ± 0.26 ^c^	4.48 ± 0.53 ^c^	5.75 ± 0.23 ^d^	ne	ne
LAB	40% CO_2_ + 60% N_2_	2.68 ± 0.10 ^a^	3.01 ± 0.28 ^a^	4.20 ± 0.35 ^a^	4.76 ± 0.48 ^a^	5.20 ± 0.31 ^a^	6.63 ± 0.13 ^a^
	60% CO_2_ + 40% N_2_	2.60 ± 0.23 ^a^	3.57 ± 0.34 ^b^	4.86 ± 0.33 ^b^	5.84 ± 0.32 ^b^	6.72 ± 0.35 ^b^	7.48 ± 0.62 ^b^
	90% CO_2_ + 10% N_2_	2.49 ± 0.12 ^a^	3.74 ± 0.42 ^b^	4.83 ± 0.42 ^b^	6.23 ± 0.63 ^c^	6.69 ± 0.37 ^b^	7.73 ± 0.21 ^b^
	Vacuum	2.46 ± 0.37 ^a^	3.20 ± 0.34 ^a^	4.16 ± 0.38 ^a^	5.59 ± 0.37 ^b^	ne	ne
H_2_S	40% CO_2_ + 60% N_2_	2.77 ± 0.35 ^a^	3.43 ± 0.54 ^a^	4.09 ± 0.20 ^a^	4.74 ± 0.11 ^a^	5.40 ± 0.32 ^a^	6.07 ± 0.53 ^a^
	60% CO_2_ + 40% N_2_	2.80 ± 0.27 ^a^	3.50 ± 0.16 ^a^	4.12 ± 0.81 ^a^	4.89 ± 0.17 ^a^	5.60 ± 0.41 ^a^	6.20 ± 0.20 ^a^
	90% CO_2_ + 10% N_2_	2.53 ± 0.38 ^b^	3.10 ± 0.20 ^b^	3.94 ± 0.24 ^a^	4.48 ± 0.52 ^b^	4.94 ± 0.24 ^b^	5.77 ± 0.28 ^b^
	Vacuum	2.57 ± 0.30 ^b^	4.83 ± 0.26 ^c^	5.48 ± 0.53 ^b^	6.73 ± 0.23 ^c^	ne	ne

Same lowercase letters in a column indicate no significant differences (*p* > 0.05). ne: not evaluated.

## Data Availability

The data presented in this study are available upon request from the corresponding authors.
